# Does azithromycin given to women in labour decrease ocular bacterial infection in neonates? A double-blind, randomized trial

**DOI:** 10.1186/s12879-017-2909-4

**Published:** 2017-12-28

**Authors:** Sarah E. Burr, Bully Camara, Claire Oluwalana, Ebrima Bojang, Christian Bottomley, Abdoulie Bojang, Robin L. Bailey, Umberto D’Alessandro, Anna Roca

**Affiliations:** 1 London School of Hygiene and Tropical Medicine, London, UK; 2 Medical Research Council Unit, The Gambia, Fajara, Banjul, The Gambia

**Keywords:** Azithromycin, Randomized trial, Neonate, Conjunctivitis, Labour

## Abstract

**Background:**

Vertical transmission can result in neonatal infection and disease. Reducing the transmission of bacterial pathogens from mother to infant may be an effective means of preventing neonatal infection, including bacterial conjunctivitis.

**Methods:**

In a double-blind, randomized trial, we assessed the effect of administering a single dose of oral azithromycin to women in labour on bacterial colonization of the neonate. A reduction in purulent neonatal conjunctivitis was a secondary objective of the trial. Ocular samples were collected from the lower fornix of infants presenting with clinical signs of purulent conjunctivitis during the first eight weeks of life. Incidence of purulent conjunctivitis was compared between trial arms. Bacterial infection was assessed using PCR and incidence of purulent conjunctivitis due to bacteria was also compared between arms.

**Results:**

Forty of 843 infants (4.7%) presented clinical signs of purulent conjunctivitis. No significant difference in incidence of purulent conjunctivitis was seen between azithromycin and placebo arms [4.3% (18/419) versus 5.2% (22/424), OR = 0.82, 95% CI (0.44,1.54), *p* = 0.628]. *S. aureus* was the most commonly identified pathogen, detected in 38% of cases. Incidence of purulent-conjunctivitis due to bacterial infection was lower in the azithromycin arm [1.2% (5/419) versus 3.8% (16/424), OR = 0.31, 95% CI (0.12–0.82), *p* = 0.025)]. The incidence of gram-positive bacteria was also lower in the azithromycin arm [1.0% (4/419) versus 3.3% (14/424), OR = 0.28, 95%CI (0.10–0.82), *p* = 0.029].

**Conclusions:**

Oral azithromycin given to women during labour may have the potential to reduce the incidence of bacterial neonatal conjunctivitis.

**Trial registration:**

ClinicalTrials.gov, identifier NCT01800942, registration date 26 Feb 2013.

## Background

Ocular bacterial infection in neonates often results from vertical transmission, from mother to child, during delivery. The most common causes are *Neisseria gonorrhoeae* and *Chlamydia trachomatis*, although infection can also be attributed to other bacteria, including *Staphylococcus aureus.* Prenatal screening and treatment of expectant mothers is an effective means of preventing neonatal infection [1, 2]. Because bacterial conjunctivitis can give rise to complications including corneal ulceration, perforation of the globe and visual impairment, many countries also recommend the use of ocular antibiotic ointment at birth as a preventive measure [3].

Vertical transmission during delivery can also result in invasive disease, including neonatal sepsis, which is an important cause of death in this age group [4]. *S. aureus* and *Streptococcus pneumoniae* are the most common cause of sepsis in African infants [5]. As asymptomatic carriage precedes bacterial invasion, interventions aimed at reducing carriage may provide an effective means of preventing disease. As an example, prenatal screening for group B streptococcus and the administration of intravenous antibiotics during labour have greatly reduced colonization and invasive neonatal disease in North American and Europe [6, 7]. However, this approach is not feasible in many low-income settings where simpler and more cost effective solutions for preventing neonatal sepsis are urgently needed. In The Gambia, West Africa, a recent randomized, double-blind trial (PregnAnZI) was conducted in which women were randomized to receive azithromycin or placebo during labour [8, 9]. Azithromycin was chosen because it can be given orally, has significant effects with a single dose and does not require transport and storage in a cold chain. The aim of the trial was to reduce maternal carriage of *S. aureus*, *S. pneumoniae* and group B streptococcus during delivery thereby preventing vertical transmission and subsequent colonization of the neonates.

Trial results indicated a significant reduction in the carriage of all study bacteria in the vaginal swabs, breast milk and nasopharyngeal samples of mothers in the azithromycin arm as compared to the placebo arm during the first four weeks following delivery. As hypothesised, bacterial carriage was also reduced in the nasopharynx of neonates whose mothers received the intervention compared to those who received placebo; this reduction was maintained throughout the first month of life [9]. A reduction in disease was also documented in mothers who received azithromycin and in their neonates indicating a clinical benefit of treatment [10].

A secondary objective of the PregnAnZI trial was to assess the effect of giving azithromycin during labour on purulent neonatal conjunctivitis. Herein, we report the incidence of purulent conjunctivitis and purulent conjunctivitis associated with bacterial infection in the trial and its aetiology. We also report a risk factors analysis for bacterial conjunctivitis among study participants.

## Methods

### Study design

This was a phase III, double-blind, placebo controlled, randomized trial. The trial protocol and results of the primary endpoints have already been published [8, 9]. Briefly, 829 women attending the study health facility during labour and who had previously given informed consent were randomized to receive an oral dose of 2 g of azithromycin or placebo. Mothers and their neonates were sampled throughout the first four weeks following delivery to determine the effect of treatment on vaginal, nasopharyngeal and breast milk carriage of *S. pneumoniae*, *S. aureus* and group B streptococcus. The primary trial end-point was nasopharyngeal carriage of the study bacteria in the neonate, at day six following delivery. As part of standard care in the country, newborns received tetracycline ointment in the eyes before hospital discharge. Active and passive follow-up was conducted for eight weeks after delivery.

### Sample collection

An ocular swab was collected from infants presenting with purulent conjunctivitis during the first eight weeks of life. Samples were collected from the lower fornix using a sterile cotton swab and kept on wet ice at the clinic. All samples were transported to the laboratory within 8 h of collection for storage at −70 °C until further processing.

### DNA extraction and PCR

DNA was extracted using the QIAamp DNA Mini Kit (QIAGEN, Germany). The presence of DNA from *S. pneumoniae*, *S. aureus*, *Moraxella catarrhalis, Haemophilus influenzae, C. trachomatis* and *N. gonorrhoeae* was assayed using the FTD SPn/Staph/MC/Hi and Vaginal Swab kits (Fast-track Diagnostics, Luxembourg) run on a RotorGene 6000 real-time PCR cycler (QIAGEN, Germany). Samples were further tested for the presence of *C. trachomatis* DNA using droplet-digital PCR according to a previously published method [11]. Laboratory staff was blinded to allocation arm.

### Outcomes

Pre-defined, secondary endpoints of the PregnAnZI trial included the proportion of newborns with at least one episode of purulent conjunctivitis and ocular *C. trachomatis* infection within the first week of life, the first four weeks of life and during the eight weeks of the follow-up period [8]. Purulent conjunctivitis caused by bacterial pathogens other than *C. trachomatis* was not a stated outcome of the trial and was investigated ad hoc.

### Statistical analysis

Incidence of purulent conjunctivitis and purulent conjunctivitis due to defined bacterial pathogens was compared between arms. Due to the small numbers of cases seen, only the endpoint at eight weeks follow-up was considered, rather than assessing the three individual time-points specified in the original protocol [8]. Odds ratios and exact *p*-values for these comparisons were calculated using the cs command in Stata. Risk factors for bacterial conjunctivitis were analysed using data from both trial arms combined and from the control arm only.

### Ethical review

The trial was approved by the Joint Gambian Government/ Medical Research Council Ethics Committee. An independent Data Safety Monitoring Board monitored the data quality and treatment safety. Written, informed consent was obtained from all women during visits to the antenatal clinic.

## Results and discussion

### Study participants

A total of 829 mothers and their offspring were recruited. Baseline characteristics were similar between intervention and control arms (Table 1).

**Table 1:** Baseline characteristics of study participants

Mothers		Azithromycin arm (*N* = 414)	Placebo arm (*N* = 415)
Characteristics		*n* (%)	*n* (%)
Age	(Median, IQR)	26.0 (22.0,30.0)	25.0 (22.0,30.0)
Ethnicity	Madinka	161 (40.1)	187 (45.8)
	Fula	77 (19.2)	64 (15.7)
	Jola	68 (17.0)	56 (13.7)
	Other	95 (23.7)	101 (24.8)
Season of delivery^a^	Rainy	141 (34.1)	143 (34.5)
Mode of delivery	Vaginal	404 (97.6)	410 (98.8)
	Caesarean	10 (2.4)	5 (1.2)
Multiple pregnancy	Yes	5 (1.2)	9 (2.2)
Hours from rupture of membrane to delivery^b^	(Median, IQR)	0.4 (0.1,1.8)	0.3 (0.1,1.3)
Newborns		Azithromycin arm (*N* = 419)	Placebo arm (*N* = 424)
Characteristics		*n* (%)	*n* (%)
Gender	Female	207 (49.4)	198 (46.7)
Apgar score at birth	0–6	14 (3.3)	11 (2.6)
	7–10	402 (96.6)	408 (97.4)
Weight^c^	(Median, IQR)	3.1 (2.8,3.5)	3.1 (2.9,3.4)
Outcomes			
Stillbirths		7 (1.7)	6 (1.4)
Deaths during the follow up period		8 (1.9)	8 (1.9)

^a^Rainy season: children born June to October

^b^Time of rupture of membranes is missing in *n* = 441 (230 in the azithromycin and 211 in the placebo arm)

^c^Weight missing in *n* = 2 (both in the placebo arm)

### Incidence of purulent conjunctivitis

Eight-hundred-forty-three babies were delivered including 13 stillbirths. Forty infants (4.7%, 40/843) presented with purulent conjunctivitis during the eight-week follow-up period. Three infants were sampled on two separate occasions (1 in the azithromycin and 2 in the placebo arm). The median age at the onset of conjunctivitis was 4 days (IQR 2 to 8). Age of onset was missing for 6 cases of conjunctivitis. The median duration of clinical signs was 7 days (IQR 4 to 9). No difference was seen in the numbers of infants presenting with purulent conjunctivitis in the azithromycin (4.3%, 18/419) versus placebo (5.2%, 22/424) arm [OR = 0.82, 95%CI (0.44–1.54), *p* = 0.628] (Table 2).

**Table 2:** Incidence of bacterial conjunctivitis reported by trial arm

Characteristic	Azithromycin arm (*N* = 419) *n* (%)	Placebo arm (*N* = 424) *n* (%)	OR (95% CI)	*p*-value
Purulent discharge	18 (4.3)	22 (5.2)	0.82 (0.44,1.54)	0.628
Any bacteria detected	5 (1.2)	16 (3.8)	0.31 (0.12,0.82)	0.025
Any gram-positive	4 (1.0)	14 (3.3)	0.28 (0.10,0.82)	0.029
*S. aureus*	4 (1.0)	12 (2.8)	0.33 (0.11,0.98)	0.074
*S. pneumoniae*	0 (0.0)	2 (0.5)	0.00 (0.00,1.94)	0.499
Any gram-negative	1 (0.2)	6 (1.4)	0.17 (0.00,1.06)	0.123
*H. influenzae*	0 (0.0)	3 (0.7)	0.00 (0.00,1.29)	0.249
*M. catarrhalis*	0 (0.0)	3 (0.7)	0.00 (0.00,1.29)	0.249
*N. gonorrhoeae*	1 (0.2)	1 (0.2)	1.01 (0.00, NA)	1.00
*C. trachomatis*	0 (0.0)	0 (0.0)	–	–
Bacterial co-infection^a^	0 (0.0)	4 (0.9)	0.00 (0.00,0.97)	0.124

^a^Bacterial co-infections: (i) *S. aureus* and *H. influenzae*; (ii) *S. pneumoniae* and *H. influenzae*; (iii) *S. aureus* and *N. gonohorreae* (iv) *M. catarrhalis*, *S. pneumoniae* and *H. influenzae*

### Aetiology

*S. aureus* was the most common bacteria, being identified in 38% (16/42) of the swabs tested (Table 2); one sample was missing in the laboratory analysis. This finding is consistent with the results of other studies in West Africa (Togo and Nigeria) that have reported *S. aureus* is a leading cause of bacterial neonatal conjunctivitis in this region [12, 13]. No significant difference was seen in the incidence of *S. aureus* infection between trial arms with an overall incidence of 1.0% (4/419) in the azithromycin and 2.8% (12/424) in the placebo arm [OR = 0.33, 95%CI (0.11–0.98), *p* = 0.074].

The incidence of infection with *S. pneumoniae*, *M. catarrhalis*, *H. influenzae* and *N. gonorrhoeae* was low. Although positive results for *S. pneumoniae*, *M. catarrhalis* and *H. influenzae* were only obtained in the placebo arm, no statistically significant difference was seen between trial arms, possibly because of the low numbers of infections detected. No sample was positive for *C. trachomatis*. While there is no recent data on urogenital *C. trachomatis* infection in The Gambia, our results are in keeping with historical data that indicates prevalence of infection is low [14, 15]. Four swabs, all in the placebo arm, showed evidence of infection with more than one bacterium; 3 co-infected with 2 bacteria and 1 with 3 bacteria. All 3 samples positive for *H. influenzae* were co-infected with another bacteria (*S. aureus*; *S. pneumoniae* and *M. catarrhalis; S. pneumoniae*).

Overall, the incidence of purulent bacterial conjunctivitis was lower in the azithromycin arm [1.2% (5/419) versus 3.8% (16/424), OR = 0.31, 95% CI (0.12–0.82), *p* = 0.025)] suggesting a beneficial effect of treatment. The incidence of conjunctivitis associated with gram-positive bacteria was also lower in the intervention arm [1.0% (4/419) versus 3.3% (14/424), OR = 0.28, 95%CI (0.10–0.82), *p* = 0.029] (Table 2). The incidence of gram-negative bacteria was lower in the intervention arm however this difference was not statistically significant [0.2% (1/419) versus 1.4% (6/424), OR = 0.17, 95% CI (0.00–1.06), *p* = 0.123].

### Risk factors

Vaginal colonization of the mother with any of the trial study bacteria (*S. aureus*, *S. pneumoniae* and group B streptococcus) prior to azithromycin administration was not a risk factor for ocular infection in the neonate (both trial arms, OR = 1.36, 95%CI 0.54–3.41, *p* = 0.468; placebo arm only, OR = 1.51, 95%CI 0.54–4.25, *p* = 0.412) (Table 3). These findings however are limited as our analysis of ocular swabs targeted more pathogens than the three study bacteria that were analysed in the vaginal swabs. Recto-vaginal swabs from mothers may also have yielded a better comparison than vaginal swabs alone.

**Table 3:** Risk factors for neonatal bacterial eye infection

Characteristic	Both trial arms				Placebo arm only			
	N	*n* (%)	OR (95%CI)	*p*-value	N	*n* (%)	OR (95%CI)	*p*-value
>18 h membrane rupture to birth								
No	384	2.3	1		205	2.9	1	
Yes	459	2.6	1.12 (0.47,2.68)	0.829	219	4.6	1.59 (0.57,4.45)	0.45
Gender								
Female	405	1.7	1		198	2.5	1	
Male	438	3.2	1.88 (0.75,4.70)	0.191	226	4.9	1.97 (0.67,5.79)	0.307
Apgar score								
0	12	0.0	NA		6	0.0	NA	
1–6	13	0.0	NA		5	0.0	NA	
7–10	810	2.6	NA	1	408	3.9	NA	1
Low birth-weight (<2.5 kg)								
No	786	2.5	1		392	3.8	1	
Yes	55	1.8	0.71 (0.09,5.39)	1	30	3.3	0.87 (0.11,6.79)	1
Bacteria in the vaginal swab prior to intervention								
No	615	2.3	1		302	3.3	1	
Yes	228	3.1	1.36 (0.54,3.41)	0.468	122	4.9	1.51 (0.54,4.25)	0.412
Season								
Dry	557	2.0	1		280	2.9	1	
Wet	286	3.5	1.80 (0.75,4.29)	0.242	144	5.6	2.00 (0.73,5.44)	0.184
Tetracycline at birth								
No	99	3.0	1		47	6.4	1	
Yes	743	2.4	0.79 (0.23,2.75)	0.728	377	3.4	0.52 (0.14,1.91)	0.404
Mothers’ years of schooling								
< 1 year	424	2.6	1		210	3.8	1	
1+ years	398	2.5	0.97 (0.41,2.30)	1	203	3.9	1.04 (0.38,2.81)	1
Ethnicity								
Madinka	352	2.3	1		189	3.7	1	0.691
Wollof	94	1.1	0.46 (0.06,3.74)		47	2.1	0.57 (0.07,4.71)	0.691
Jola	127	5.5	2.51 (0.89,7.06)		59	6.8	1.89 (0.53,6.70)	0.691
Fula	144	2.8	1.23 (0.36,4.15)		66	4.5	1.24 (0.31,4.93)	0.691
Other	106	0.9	0.41 (0.05,3.31)	0.23	56	1.8	0.47(0.06,3.93)	0.691

Differences in risk of neonatal bacterial conjunctivitis were not significant among infants who received tetracycline ointment in the eye at birth compared to those who did not although odds ratios were lower for those who received ointment (both trial arms, OR = 0.79, 95%CI 0.23–2.75, *p* = 0.728; placebo arm only, OR = 0.52, 95%CI 0.14–1.91, *p* = 0.404) (Table 3). While other topical antimicrobial agents are available for use in neonates, evidence suggests that prophylaxis with tetracycline ointment does result in better outcomes in comparison to others such as povidone-iodine or erythromycin [16, 17]. Premature rupture of the membranes, which is a risk factor for conjunctivitis in the low-income setting of Malawi [18], was not a risk factor in The Gambia (both trial arms, OR = 1.12, 95%CI 0.47–2.68, *p* = 0.829; placebo arm only, OR = 1.59, 95%CI 0.57–4.45, *p* = 0.450) (Table 3).

### Limitations

The majority of infants in the trial received ocular tetracycline ointment after delivery, as is standard care in The Gambia. This may have lowered incidence of infection or modified the distribution of pathogens thereby impacting our endpoint. Our study is also limited by statistical power, as the trial was not designed to detect differences in the incidence of conjunctivitis or bacterial conjunctivitis between study arms. While the results showed a trend of lower incidence in the azithromycin arm for almost all bacteria assayed, significant results were obtained only when grouping all purulent bacterial conjunctivitis and conjunctivitis associated with gram-positive bacteria. The majority of samples collected from the azithromycin arm were negative for all the bacteria assayed suggesting conjunctivitis in these infants may have been chemical or viral in nature. However, our panel focused only on the major causes of bacterial conjunctivitis in neonates. It is possible that some cases could have been due to less common bacteria, yet there is no reason to believe that any other bacteria would have been more prevalent in the azithromycin arm. The limited power also extends to the risk factor analysis and may explain why tetracycline ointment or the premature rupture of membranes were not associated with conjunctivitis.

Ocular swabs were collected in the absence of transport media for the detection of *C. trachomatis* DNA by molecular means, as purulent conjunctivitis with *C. trachomatis* infection was a pre-specified outcome of the trial. This prevented us from evaluating other bacterial infection by routine culture. However, PCR is generally considered more sensitive than conventional bacteriological techniques so we do not anticipate this adversely impacted our results.

## Conclusions

While numbers were small, our data suggest azithromycin given in labour may provide a means of decreasing the risk of ocular bacterial infection in the neonate. Larger scale trials to determine the impact of azithromycin treatment in labour on neonatal sepsis are currently underway. If these studies show a benefit, there may be a case for recommending this intervention for deliveries as a means of decreasing neonatal mortality.

## Abbreviations

PregnAnZI: Prevention of bacterial infections in newborn trial
